# Association between the Non-high-Density Lipoprotein Cholesterol to High-Density Lipoprotein Cholesterol Ratio and the Risk of Coronary Artery Disease

**DOI:** 10.1155/2020/7146028

**Published:** 2020-03-07

**Authors:** Jiayin You, Zhenhao Wang, Guoping Lu, Zhenyue Chen

**Affiliations:** Department of Cardiology, Ruijin Hospital, Shanghai Jiao Tong University School of Medicine, Shanghai, China

## Abstract

**Background:**

The purpose of this study was to evaluate the association between the non-high-density lipoprotein cholesterol (non-HDL-C) to high-density lipoprotein cholesterol (HDL-C) ratio and the risk of coronary artery disease (CAD). We also explored the potential role of non-HDL-C/HDL-C in the prognosis of CAD.

**Methods:**

We analyzed 930 consecutive patients with chest discomfort who underwent coronary angiography. Of these, 680 were diagnosed with CAD; the remaining 250 patients were normal. Multivariate logistic regression and receiver operating characteristic (ROC) curves were employed to evaluate the association between non-HDL-C/HDL-C and CAD. The effect of non-HDL-C/HDL-C on the progression of major adverse cardiovascular events (MACEs) was also explored.

**Results:**

Increased non-HDL-C/HDL-C was associated with an increased risk of CAD (OR: 1.291; 95% CI: 1.039-1.561; *P* = 0.013). The results of stratified analyses by CAD subtype showed a correlation between high non-HDL-C/HDL-C and increased risk of acute coronary syndrome (OR: 1.661; 95% CI: 1.259-2.207; *P* = 0.013). The results of stratified analyses by CAD subtype showed a correlation between high non-HDL-C/HDL-C and increased risk of acute coronary syndrome (OR: 1.661; 95% CI: 1.259-2.207; *P* = 0.013). The results of stratified analyses by CAD subtype showed a correlation between high non-HDL-C/HDL-C and increased risk of acute coronary syndrome (OR: 1.661; 95% CI: 1.259-2.207; *P* = 0.013). The results of stratified analyses by CAD subtype showed a correlation between high non-HDL-C/HDL-C and increased risk of acute coronary syndrome (OR: 1.661; 95% CI: 1.259-2.207; *P* = 0.013). The results of stratified analyses by CAD subtype showed a correlation between high non-HDL-C/HDL-C and increased risk of acute coronary syndrome (OR: 1.661; 95% CI: 1.259-2.207;

**Conclusions:**

The findings of this study indicated that non-HDL-C/HDL-C plays an important role in the risk and progression of CAD. These findings need verification by further large-scale prospective studies.

## 1. Introduction

Coronary artery disease (CAD) is one of the most common diseases that endanger global human health, and its incidence has increased over the years [1, 2]. Several studies have already recognized that dyslipidaemia is significantly associated with the risk of atherosclerosis (AS) and that low-density lipoprotein cholesterol (LDL-C) is the most important criterion in the serum lipid profile. Therefore, LDL-C is currently the first target of lipid-lowering therapy in patients with CAD [3]. However, the incidence of cardiovascular disease (CVD) in CAD patients is still high although the level of LDL-C is effectively reduced in these patients [4–6].

Non-high-density lipoprotein cholesterol (non-HDL-C) refers to the sum of cholesterol in lipoproteins other than high-density lipoprotein cholesterol (HDL-C). Studies have already found that non-HDL-C is a strong independent predictor for the risk of CVD, which should be considered to be a secondary target of lipid-lowering therapy in atherosclerotic cardiovascular disease or in patients at high risk [3, 7, 8]. More recently, the non-HDL-C to HDL-C ratio (non-HDL-C/HDL-C) has attracted increasing attention, and this is significantly correlated with the metabolic syndrome [9]. A study conducted by Zhao et al. indicated that non-HDL-C/HDL-C was superior to traditional lipid profiles for evaluating the degree of AS [10]. However, there are few studies that have looked at the association of non-HDL-C/HDL-C with the risk and prognosis of CAD. Therefore, the current study was conducted to evaluate the association between non-HDL-C/HDL-C and the risk of CAD. The potential effect of non-HDL-C/HDL-C on the risk of major adverse cardiovascular events (MACEs) in patients with CAD was also explored.

## 2. Patient and Methods

### 2.1. Study Population

A total of 930 consecutive patients (545 men and 385 women) aged 33-96 years with chest discomfort who underwent coronary angiography at Ruijin Hospital affiliated to Shanghai Jiao Tong University School of Medicine between August 2008 and August 2010 were retrospectively recruited. Patients diagnosed with chronic heart failure (NHHA III-IV), myocarditis, cardiomyopathy, valvular heart disease, myocardial bridge, pericardial effusion, cor pulmonale, myocardial infarction caused by other causes, diseases that cause acute anxiety (such as acute abdomen, cerebral haemorrhage, and acute phase of cerebral infarction), severe hepatic or renal dysfunction, thyroid diseases, tumours, systemic immune diseases, and acute and chronic infections were excluded. Of 930 individuals, 680 patients had CAD (CAD group), which was defined as stenosis ≥ 50% of at least one major vessel (left main artery, left anterior descending artery, left circumflex artery, and/or right coronary artery) by coronary angiography. The remaining 250 patients showed normal coronary arteries without significant stenosis (<50%) by coronary angiography (normal group). Patients in the CAD group were further divided into stable angina (SA, 339 patients with typical symptoms of exertional angina and in whom coronary angiography showed ≥50% stenosis of at least one major vessel) and acute coronary syndrome (ACS, 341 patients who had at least two of the following three criteria: (I) clinical history of ischemic chest pain, (II) dynamic evolution of electrocardiogram, and (III) dynamic evolution of myocardial enzymes). Moreover, 266 patients in the CAD group were diagnosed with multivessel disease (MVD) or left main disease.

### 2.2. Collected Data and Laboratory Tests

The baseline characteristics of recruited patients, including general information (sex, age, body mass index (BMI), smoking, hypertension, hyperlipidaemia, and diabetes) and laboratory tests (triglyceride (TG), total cholesterol (TC), LDL-C, HDL-C, fasting blood glucose (FBG), high-sensitivity C-reactive protein (hsCRP), N-terminal-proB-type natriuretic peptide (NT-proBNP), creatine kinase (CK), CK-MB (CK-muscle/brain), and troponin I (TnI)), and the use of statins were recorded. In addition, in the CAD, ACS, and SA groups, the quartiles of the non-HDL-C/HDL-C were calculated. The cutoff values of non-HDL-C/HDL-C for CAD were 2.251, 3.077, and 4.005, respectively, the cutoff values of non-HDL-C/HDL-C for ACS were 2.458, 3.250, and 4.074, respectively, and the cutoff values of non-HDL-C/HDL-C for SA were 2.049, 2.822, and 3.770, respectively (25^th^, 50^th^, and 75^th^ percentiles, respectively, in each case).

### 2.3. Coronary Angiography and Severity of CAD

Coronary angiography was performed using the Judkins technique with INNOVA 20000 equipment (GE Healthcare, Waukesha, Wis.), by qualified interventional cardiologists, following the American College of Cardiology and the American Heart Association (ACC/AHA) guidelines for coronary angiography. Coronary angiograms were evaluated by two experienced interventional cardiologists who were unaware of the patients' disease status. The patients were divided into single-vessel disease (SVD), double-vessel disease (DVD), and three-vessel disease according to the number of branch vessels involved, among which three-vessel disease and left main disease were classified as MVD. The Gensini score was used to measure the severity of coronary atherosclerosis [11]. This method categorizes narrowing of the lumen of the coronary arteries as 1 = 1 − 25% stenosis, 2 = 26 − 50% stenosis, 4 = 51 − 75% stenosis, 8 = 76 − 90% stenosis, and 32 = total occlusion. The score is then multiplied by a factor that represents the importance of the location of the lesions in the coronary arterial system: for example, 5 for the left main coronary artery, 2.5 for the proximal left anterior descending or the proximal left circumflex artery, 1.5 for the midregion, and 1 for the distal left anterior descending or middistal region of the left circumflex artery. The sum of all coronary artery disease scores is the Gensini score. The higher the score, the more severe the coronary artery lesions. Patients with CAD were divided into three groups according to the tertiles of the Gensini score (low Gensini score: ≤19, intermediate Gensini score: 19-43, and high Gensini score: >43).

### 2.4. Follow-Up

Clinical follow-up data regarding participating patients were obtained either through an outpatient follow-up or by telephone contact after discharge. The patients with CAD were further divided into four groups according to non-HDL-C/HDL-C quartiles and were compared among the groups for the incidence of MACEs. MACEs were defined as frequent occurrence of angina, new onset or recurrence of myocardial infarction, unexpected coronary revascularization, new onset or recurrence of heart failure, arrhythmia with compromised hemodynamic function, cerebrovascular accident, and death from any cause. The median follow-up duration in this study was 7.51 (±0.57) years and involved 673 CAD patients.

### 2.5. Statistical Analysis

Continuous variables are expressed as mean ± standard deviation (mean ± SD), and categorical variables are expressed as the number of cases and their percentages (*n* (%)). Parametric variables were compared between groups by using Student's *t*-test, chi-squared test, and one-way analysis of variance (ANOVA). Nonparametric variables were compared between groups by using Dennett's T3 method. Pearson's correlation analyses were employed to explore the relationship between serum lipid and inflammation parameters and Gensini scores. Multivariate logistic regression analyses were conducted to evaluate the association of non-HDL-C/HDL-C with the risk of CAD, ACS, high Gensini score, and MVD. The receiver operating characteristic (ROC) curves were employed to evaluate the diagnostic value of non-HDL-C/HDL-C for CAD, ACS, high Gensini score, and MVD. All the *P* values reported were two-tailed, and values less than 0.05 were considered to be statistically significant. The above statistical analyses were performed using SPSS 23.0 (SPSS Inc., Chicago, Ill.). The figures were created using GraphPad Prism version 6.0c.

## 3. Results

### 3.1. Baseline Characteristics

A total of 930 consecutive patients with chest discomfort admitted to our hospital were recruited and consisted of 545 men and 385 women. Data regarding in-hospital outcomes were available for 930 patients (100%), and 7.51 ± 0.57 − year follow-up data were available for 819 patients (88.06%). Coronary angiography showed that 250 patients had no obvious stenosis, and they were defined as the normal group; the remaining 680 patients were defined as the CAD group. There were significant differences between the CAD and the normal groups in sex, age, smoking, hypertension, diabetes, HDL-C, non-HDL-C, non-HDL-C/HDL-C, hsCRP, TnI, CK-MB, pro-BNP, and the use of statins (*P* < 0.05). Moreover, the differences for sex, age, hypertension, hyperlipidaemia, diabetes HDL-C, non-HDL-C/HDL-C, hsCRP, and the use of statins between the SA and the normal groups were statistically significant (*P* < 0.05). Furthermore, there were significant differences between the ACS and the normal groups for sex, age, smoking, diabetes, LDL-C, HDL-C, non-HDL-C/HDL-C, hsCRP, TnI, CK-MB, pro-BNP, and the use of statins (*P* < 0.05). Finally, we noted significant differences between ACS and SA for sex, smoking, hypertension, diabetes, LDL-C, HDL-C, non-HDL-C/HDL-C, hsCRP, TnI, CK-MB, and pro-BNP (*P* < 0.05) (Table 1).

According to coronary angiography, patients in the CAD group can be further divided into the SVD group, the DVD group, and the MVD group. Compared with the normal group, patients in the DVD and MVD groups were significantly older and had significantly higher proportions of males, smoking history, hypertension, diabetes, and the use of statins. LDL-C, non-HDL-C, non-HDL-C/HDL-C, and hsCRP values were significantly higher in the MVD group than in the normal group. The details of patient characteristics are presented in Table 2.

### 3.2. Non-HDL-C/HDL-C and the Severity of CAD

**Figure 1:**
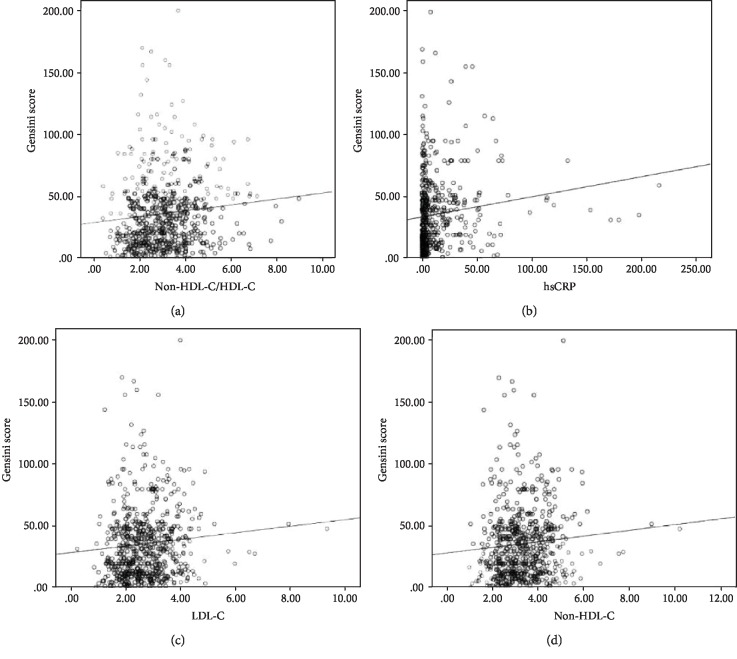
Scatter diagrams indicated the correlation of non-HDL-C/HDL-C and (a) hsCRP, (b) LDL-C, (c) non-HDL-C, and (d) Gensini score.

**Figure 2:**
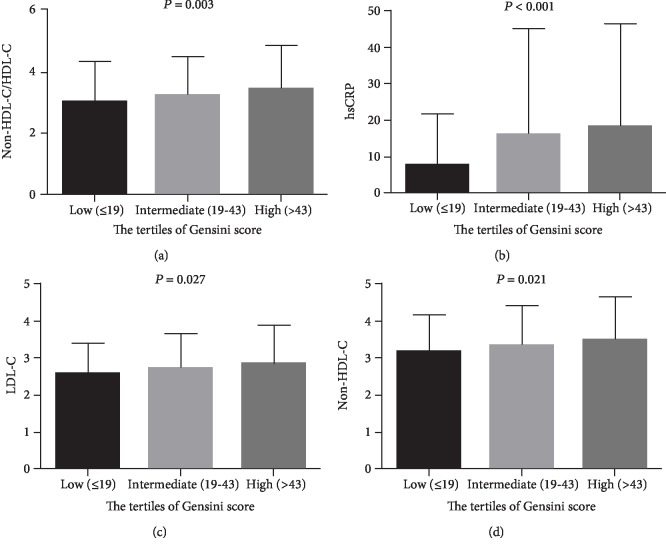
Comparison of non-HDL-C/HDL-C (a), hsCRP (b), LDL-C (c), and non-HDL-C (d) levels among tertile groups of the Gensini score.

Pearson's correlation analysis was employed to evaluate the association of serum lipid and inflammation indices with the Gensini scores, setting the Gensini scores as the dependent variable and TC, LDL-C, non-HDL-C, non-HDL-C/HDL-C, and hsCRP as independent variables. We noted that non-HDL-C/HDL-C, hsCRP, LDL-C, and non-HDL-C had a positive correlation with the Gensini scores (*r* was 0.107, 0.136, 0.085, and 0.084, respectively, *P* < 0.05 for all, Figure 1). Moreover, there were significant differences between these parameters and the tertiles of the Gensini scores (*P* < 0.05 for all), and patients in the upper tertile of the Gensini scores had significantly higher levels of non-HDL-C/HDL-C, hsCRP, LDL-C, and non-HDL-C compared with the lower tertile of GS (Figure 2).

### 3.3. Non-HDL-C/HDL-C and CAD

**Figure 3:**
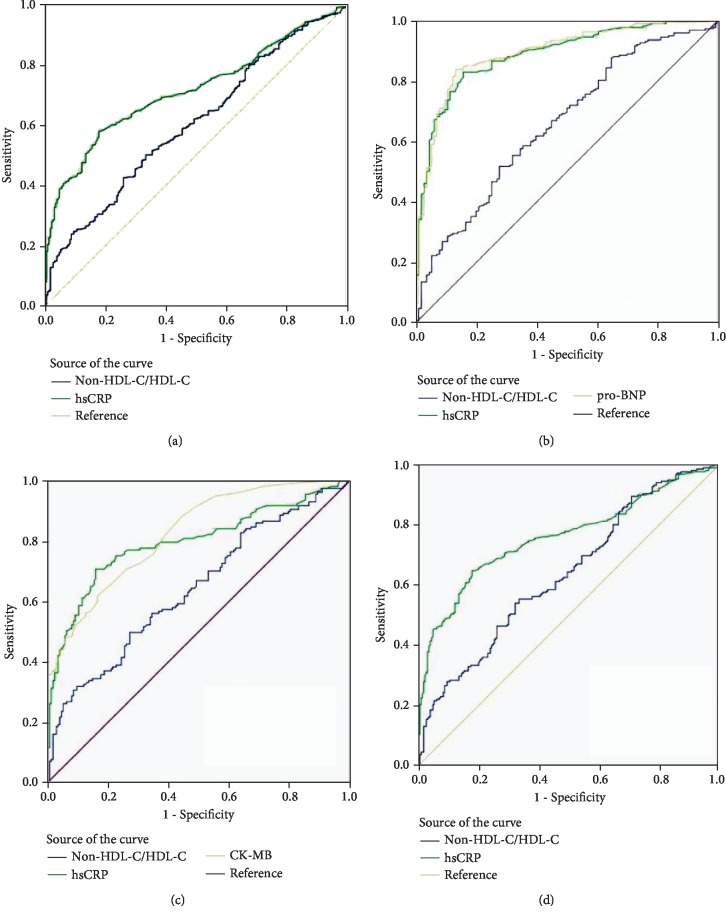
ROC curve for the diagnosis of CAD (a), ACS (b), high GS (c), and MVD (d).

The multivariate logistic regression analysis indicated that a higher non-HDL-C/HDL-C value was associated with an increased risk of CAD after adjusting for gender, age, history of smoking, hypertension, diabetes, and the use of statins (OR: 1.291; 95% CI: 1.039-1.561; *P* = 0.013). When stratified by CAD subtypes, increased non-HDL-C/HDL-C was correlated with greater risk of ACS (OR: 1.661; 95% CI: 1.259-2.207; *P* < 0.001), high Gensini score (OR: 1.408; 95% CI: 1.021-1.935; *P* = 0.039), and MVD (OR: 1.487; 95% CI: 1.128-1.992; *P* = 0.007) (Table 3). In addition, the area under the ROC curve (AUC) was employed to assess the diagnostic value of blood parameters that were associated with CAD, ACS, high GS, and MVD. The AUC of non-HDL-C/HDL-C was 0.604 (95% CI: 0.562-0.646; *P* < 0.001), and the best cutoff value for the diagnosis of CAD was 3.092, with sensitivity of 50.2% and specificity of 66.7%. The AUC of non-HDL-C/HDL-C was 0.658 (95% CI: 0.597-0.719; *P* < 0.001), and the best cutoff value of non-HDL-C/HDL-C for the diagnosis of ACS was 3.345, with sensitivity of 51.6% and specificity of 72.4%. Furthermore, the AUC of non-HDL-C/HDL-C was 0.642 (95% CI: 0.583-0.702; *P* < 0.001), and the best cutoff value of non-HDL-C/HDL-C for the diagnosis of high Gensini scores was 3.338, with sensitivity of 49.7% and specificity of 72.9%. Finally, the AUC of non-HDL-C/HDL-C was 0.636 (95% CI: 0.585-0.686; *P* < 0.001), and the best cutoff value of non-HDL-C/HDL-C for the diagnosis of MVD was 3.092, with sensitivity of 55.2% and specificity of 66.7% (Figure 3).

### 3.4. Non-HDL-C/HDL-C and MACEs in CAD Patients

**Figure 4:**
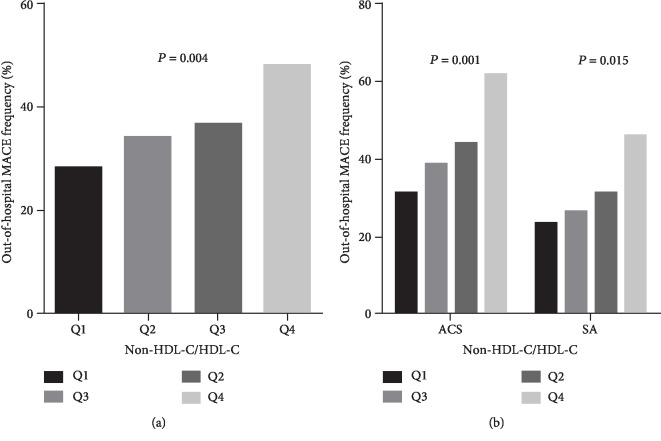
Comparison of incidence rate of out-of-hospital MACEs in patients with CAD (a) and ACS and SA (b) between interquartile groups of non-HDL-C/HDL-C.

A total of 222 MACEs (27.11%) occurred during 7.51 years of follow-up, which comprised 23 out-of-hospital deaths, 67 cases of frequent occurrence of angina, 29 cases of new onset or recurrence of myocardial infarction, 15 cases of arrhythmia with compromised hemodynamic function, 57 cases of new onset or recurrence of heart failure, and 31 cases of cerebrovascular accident. Overall, we noted that an increased non-HDL-C/HDL-C was associated with excess risk of MACEs in CAD patients (*P* = 0.004), ACS patients (*P* = 0.001), and SA patients (*P* = 0.015) (Figure 4).

## 4. Discussion

The primary findings of our study were that non-HDL-C/HDL-C was associated with an increased risk of CAD, ACS, high Gensini score, and MVD. Moreover, the area under the ROC curves indicated that the predictive value of serum non-HDL-C/HDL-C for CAD, ACS, high Gensini score, and MVD was weak. Furthermore, the incidence of MACEs was significantly increased in CAD patients with high non-HDL-C/HDL-C. Similar conclusions were reached for ACS and SA.

Previous studies have already found that dyslipidaemia was a well-documented risk factor for CAD and that elevated LDL-C level played a critical role in the pathogenesis of AS [12]. Moreover, non-HDL-C as a kind of lipoprotein cholesterol that includes LDL-C, very low-density lipoprotein cholesterol (VLDL-C), intermediate-density lipoprotein (IDL) cholesterol, Lp (a), and chylomicrons was a clear-cut risk factor, especially in patients with hypertriglyceridaemia. The use of non-HDL-C/HDL-C takes into account the influence of both non-HDL-C and HDL-C, so it is speculated that it has a better correlation with CAD and the severity of coronary lesions than either of the subfractions alone. Numerous studies have already investigated the role of lipid and lipoprotein ratios in the severity and prognosis of coronary artery lesions [13–18]. However, most studies focused on patients with chronic SA, and the potential role of lipid and lipoprotein ratios in the prognosis of disease was not investigated in these studies. Therefore, in the current study, both SA and ACS patients were recruited, and the association of non-HDL-C/HDL-C with the risk and prognosis of CAD was evaluated. The possible difference in association according to CAD subtypes was also explored.

The current study indicated that increased non-HDL-C/HDL-C was associated with an increased risk of CAD and ACS, high Gensini score, and MVD. The reason for this could be that most CAD patients had an elevated non-HDL-C level and decreased HDL-C level, which may increase the propensity to lead to atherosclerotic plaque formation in the coronary arteries. The association between non-HDL-C/HDL-C and the severity of coronary artery lesions represented by Gensini score and MVD, which indicated that high non-HDL-C/HDL-C is an independent predictor of severe coronary artery lesions, and the potential reasons for these have already been illustrated in numerous previous studies [13, 15, 17, 18]. Therefore, non-HDL-C/HDL-C is not only associated with an increased risk of CAD but also related to the progression of AS. However, we did not find any significant association between non-HDL-C/HDL-C and the risk of SA. The reason for this could be that most patients with known SA are likely to be taking lipid-lowering drugs.

A previous study indicated that a high apoB/apoA1 ratio and high non-HDL-C levels are significantly correlated with in-hospital MACEs and out-of-hospital adverse endpoints, including angina, myocardial infarction, new-onset heart failure, stroke, and cardiac death [13]. However, no researchers have investigated the effect of non-HDL-C/HDL-C on the risk of long-term adverse endpoints. This study found that increased non-HDL-C/HDL-C might play an important role in the progression of MACE in the long-term in CAD patients, and this result was also evident in the SA and the ACS groups. However, any influence of potential confounding factors was not fully evaluated, so the optimal validity of our results needs to be verified by large-scale prospective studies.

Several limitations of this study should be highlighted: (1) this study used a retrospective design, which makes it less reliable than a prospective design; (2) all the recruited patients were from a single hospital, which might have introduced selection bias stemming from the limited sample size; (3) the background uses of hypoglycemic and hypotensive drugs were not collected, which could have modified the progression and prognosis of CAD; and (4) the severity of coronary artery lesions was assessed by using the Gensini score, which might be inferior to the SYNTAX score for assessing the severity of coronary artery lesions.

## 5. Conclusions

The findings of this study indicated that serum non-HDL-C/HDL-C is significantly associated with the progression and severity of CAD. Moreover, the incidence of MACE in the long term was significantly increased in CAD patients with high non-HDL-C/HDL-C, irrespective to the presence of SA or ACS. Further prospective studies in large cohorts are warranted to validate the findings of the present study.

## Figures and Tables

**Table 1:** Patient characteristics.

Variables	Normal group (*N* = 250)	CAD group (*N* = 680)	SA group (*N* = 339)	ACS group (*N* = 341)
Male (*n* (%))	110 (44.0%)	435 (64.0%)^∗∗∗^	177 (52.2%)^∗^	259 (75.7%)^∗∗∗†††^
Age (years)	60.86 ± 9.69	66.55±11.13^∗∗∗^	66.45±9.82^∗∗∗^	66.67±12.29^∗∗∗^
BMI (kg/m^2^)	24.95 ± 3.45	24.41 ± 3.86	24.54 ± 2.87	24.29 ± 4.64
Smoking (*n* (%))	67 (36.6%)	282 (41.5%)^∗∗∗^	101 (29.8%)	182 (53.2%)^∗∗∗†††^
Hypertension (*n* (%))	153 (61.2%)	482 (70.9%)^∗∗^	255 (75.2%)^∗∗∗^	227 (66.4%)^†^
Hyperlipidaemia (*n* (%))	35 (14.0%)	88 (12.9%)	44 (13.1%)^∗∗∗^	44 (12.9%)
Diabetes [*n* (%)]	55 (22.0%)	282 (41.5%)^∗∗∗^	181 (53.4%)^∗∗∗^	101 (29.5%)^∗†††^
TC (mmol/L)	4.36 ± 0.95	4.44 ± 1.08	4.35 ± 1.15	5.67 ± 22.09
LDL-C (mmol/L)	2.57 ± 0.80	2.69 ± 0.94	2.59 ± 0.96	2.79±0.91^∗∗††^
HDL-C (mmol/L)	1.22 ± 0.32	1.11±0.31^∗∗∗^	1.14±0.32^∗∗^	1.08±0.30^∗∗∗††^
Non-HDL-C (mmol/L)	3.15 ± 0.89	3.31 ± 1.08^∗^	3.21 ± 1.13	4.58 ± 22.05
Non-HDL/HDL	2.76 ± 1.04	3.20±1.32^∗∗∗^	3.04±1.35^∗∗^	3.36±1.28^∗∗∗††^
hsCRP (mg/L)	2.48 ± 4.51	12.13±22.21^∗∗∗^	4.07±6.62^∗∗^	25.17±31.71^∗∗∗†††^
TnI (*μ*g/L)	0.02 ± 0.06	10.87±26.40^∗∗∗^	0.16 ± 1.93	20.97±33.74^∗∗∗†††^
CK-MB (U/L)	1.35 ± 1.02	34.79±80.58^∗∗∗^	2.58 ± 12.01	65.28±102.76^∗∗∗†††^
Pro-BNP (fmol/mL)	249.31 ± 959.85	1960.34±4251.81^∗∗∗^	431.94 ± 1115.81	3007.99±5193.59^∗∗∗†††^
Users of statins (%)	67 (26.8%)	322 (47.4%)^∗∗∗^	189 (55.8%)^∗∗∗^	133 (39.0%)^∗∗∗^

CHD: coronary artery disease; SA: stable angina; ACS: acute coronary syndrome; BMI: body mass index; TC: total cholesterol; LDL-C: low-density lipoprotein cholesterol; HDL-C: high-density lipoprotein cholesterol; non-HDL-C: non-high-density lipoprotein cholesterol; non-HDL/HDL: non-high-density lipoprotein cholesterol to high-density lipoprotein cholesterol ratio; hsCRP: high-sensitivity C-reactive protein; TnI: troponin I; CK-MB: creatine kinase isoenzyme; pro-BNP: brain natriuretic peptide. Compared with the normal group: ^∗^*P* < 0.05, ^∗∗^*P* < 0.01, and ^∗∗∗^*P* < 0.001. ACS group versus SA group: ^†^*P* < 0.05, ^††^*P* < 0.01, and ^†††^*P* < 0.001.

**Table 2:** Patient characteristics.

Variables	Normal group (*N* = 250)	SVD group (*N* = 188)	DVD group (*N* = 184)	MVD group (*N* = 266)
Male (*n* (%))	110 (44.0%)	99 (52.7%)	118 (64.1%)^∗∗∗†^	192 (72.2%)^∗∗∗†††^
Age (years)	60.86 ± 9.69	64.15±11.32^∗∗^	65.77±10.55^∗∗∗^	68.18±10.76^∗∗∗†††^
BMI (kg/m^2^)	24.95 ± 3.45	24.23 ± 3.03^∗^	24.49 ± 2.95	24.64 ± 4.67
Smoking (*n* (%))	67 (36.6%)	75 (39.9%)^∗∗^	89 (48.4%)^∗∗∗^	102 (38.3%)^∗∗^
Hypertension (*n* (%))	153 (61.2%)	126 (67.0%)	138 (75.0%)^∗∗^	187 (70.8%)^∗^
Hyperlipidaemia (*n* (%))	35 (14.0%)	29 (15.4%)	26 (14.1%)	24 (9.1%)^†^
Diabetes (*n* (%))	55 (22.0%)	78 (41.5%)^∗∗∗^	68 (37.0%)^∗∗^	113 (42.5%)^∗∗∗^
TC (mmol/L)	4.36 ± 0.95	4.36 ± 1.15	4.34 ± 1.10	4.48 ± 1.07
LDL-C (mmol/L)	2.57 ± 0.80	2.63 ± 0.99	2.64 ± 0.94	2.76 ± 0.90^∗^
HDL-C (mmol/L)	1.22 ± 0.32	1.15 ± 0.37^∗^	1.10±0.29^∗∗∗^	1.08±0.29^∗∗∗†^
Non-HDL-C (mmol/L)	3.15 ± 0.89	3.21 ± 1.11	3.23 ± 1.09	3.40±1.02^∗∗^
Non-HDL/HDL	2.76 ± 1.04	3.04 ± 1.28^∗^	3.15±1.37^∗∗^	3.33±1.27^∗∗∗†^
hsCRP (mg/L)	2.48 ± 4.51	12.07±23.44^∗∗∗^	12.03±18.20^∗∗∗^	17.31±30.09^∗∗∗†^
TnI (*μ*g/L)	0.02 ± 0.06	9.50±24.40^∗∗∗^	8.55±24.21^∗∗∗^	12.59±28.27^∗∗∗^
CK-MB (U/L)	1.35 ± 1.02	36.04±82.36^∗∗∗^	33.14±82.14^∗∗∗^	32.59±75.07^∗∗∗^
Pro-BNP (fmol/mL)	249.31 ± 959.85	1370.66±2787.54^∗∗∗^	1096.83±1552.57^∗∗∗^	2341.45±4984.23^∗∗∗†^
Users of statins (%)	67 (26.8%)	87 (46.3%)^∗∗∗^	91 (49.5%)^∗∗∗^	130 (48.9%)^∗∗∗^

SVD: single-vessel disease; DVD: double-vessel disease; MVD: multivessel disease; TC: total cholesterol; LDL-C: low-density lipoprotein cholesterol; HDL-C: high-density lipoprotein cholesterol; non-HDL-C: non-high-density lipoprotein cholesterol; non-HDL/HDL: non-high-density lipoprotein cholesterol to high-density lipoprotein cholesterol ratio; hsCRP: high-sensitivity C-reactive protein; TnI: troponin I; CK-MB: creatine kinase isoenzyme; pro-BNP: brain natriuretic peptide. Compared with the normal group: ^∗^*P* < 0.05, ^∗∗^*P* < 0.01, and ^∗∗∗^*P* < 0.001. Compared with the SVD group: ^†^*P* < 0.05, ^††^*P* < 0.01, and ^†††^*P* < 0.001.

**Table 3:** Multivariate logistic regression analysis for the association of non-HDL-C/HDL-C with CAD and specific CAD subtypes.

Outcomes	*P*	OR	95% CI	
			Lower	Upper
CAD	0.013	1.291	1.039	1.561
ACS	<0.001	1.661	1.259	2.207
High Gensini score	0.039	1.408	1.021	1.935
MVD	0.007	1.487	1.128	1.992

^∗^Adjusted for gender, age, diabetes, hypertension, smoking, and the use of statins.

## Data Availability

All data generated or analyzed during this study are included in this published article.
